# Optimization of β-1,4-Endoxylanase Production by an *Aspergillus niger* Strain Growing on Wheat Straw and Application in Xylooligosaccharides Production

**DOI:** 10.3390/molecules26092527

**Published:** 2021-04-26

**Authors:** Zahra Azzouz, Azzeddine Bettache, Nawel Boucherba, Alicia Prieto, Maria Jesus Martinez, Said Benallaoua, Laura Isabel de Eugenio

**Affiliations:** 1 Laboratoire de Microbiologie Appliquée (LMA), Faculté des Sciences de la Nature et de la Vie, Université de Bejaia, 06000 Bejaia, Algeria; zahraazzouz@yahoo.fr (Z.A.); bettache84@hotmail.fr (A.B.); boucherbanawel@yahoo.fr (N.B.); 2 Biotechnology for Lignocellulosic Biomass Group, Centro de Investigaciones Biológicas (CIB-CSIC), C/Ramiro de Maeztu 9, 28040 Madrid, Spain; aliprieto@cib.csic.es (A.P.); mjmartinez@cib.csic.es (M.J.M.)

**Keywords:** fungi, enzymes, hemicellulose, xylan, wastes, hydrolysis

## Abstract

Plant biomass constitutes the main source of renewable carbon on the planet. Its valorization has traditionally been focused on the use of cellulose, although hemicellulose is the second most abundant group of polysaccharides on Earth. The main enzymes involved in plant biomass degradation are glycosyl hydrolases, and filamentous fungi are good producers of these enzymes. In this study, a new strain of *Aspergillus niger* was used for hemicellulase production under solid-state fermentation using wheat straw as single-carbon source. Physicochemical parameters for the production of an endoxylanase were optimized by using a One-Factor-at-a-Time (OFAT) approach and response surface methodology (RSM). Maximum xylanase yield after RSM optimization was increased 3-fold, and 1.41- fold purification was achieved after ultrafiltration and ion-exchange chromatography, with about 6.2% yield. The highest activity of the purified xylanase was observed at 50 °C and pH 6. The enzyme displayed high thermal and pH stability, with more than 90% residual activity between pH 3.0–9.0 and between 30–40 °C, after 24 h of incubation, with half-lives of 30 min at 50 and 60 °C. The enzyme was mostly active against wheat arabinoxylan, and its kinetic parameters were analyzed (*K_m_* = 26.06 mg·mL^−1^ and *V_max_* = 5.647 U·mg^−1^). Wheat straw xylan hydrolysis with the purified β-1,4 endoxylanase showed that it was able to release xylooligosaccharides, making it suitable for different applications in food technology.

## 1. Introduction

Each year, transformation of the animal and plant feedstock, and their intermediate and terminal products, yields 140 billion tons of agricultural and industrial residues worldwide. Just 3% of the 13 billion tons/year of plant-biomass residues are utilized in producing goods [1]. The current management system is highly dependent on various fossil energy sources like oil, coal and natural gas [2]. The development of technologies for renewable energy production is one of the greatest challenges in the twenty-first century, due to the serious problems of energy production and use [3]. Alternative energy sources like biomass, solar, wind and hydrothermal energy can alter fossil fuels needs in the future. Among these, biomass is one of the carbon-based sustainable energy sources most used in the world [4].

Agriculture wastes are frequently rich in nutrients, and can provide an excellent habitat for the development of microorganisms. Lignocellulose, the main renewable resource in nature and the most abundant organic material in biosphere, is the central component of plant biomass. It consists of three principal constituents: (i) cellulose, a homopolysaccharide composed of d-glucopyranose units linked by β-1,4-*O*-glycosidic bonds, constituting long linear chains which interact with each other giving rise to microfibrils [5]; (ii) hemicellulose, which groups a series of polysaccharides with heterogeneous structures, that differ from each other in their backbone and branches [6]; and (iii) lignin, an amorphous and complex heteropolymer, highly branched and composed by phenylpropane units, which reinforces plant cell wall structure [7].

In hemicellulose, xylan is the most abundant non-cellulosic plant polysaccharide, being the main component in the secondary cell wall of dicotyledons and cereal grains [8]. It is composed of a backbone of β-1,4-linked d-xylopyranosyl units, frequently acetylated and highly branched by short side chains of arabinose or glucuronic acid. The abundance of each of these substituents depends largely on the nature of the plant biomass. Usually, full catabolic processes are achieved in nature by the synergistic action of different microorganisms found in biomass-rich environments [9]. However, during last years, the studies about the enzymatic microbial system involved in the process have increased for the economical relevance of exploiting this heteropolysaccharide on different biotechnological applications. Due to its complexity, xylan hydrolysis requires the action of different enzymes for its degradation but, among them, two types of glycosidases play a key role in the process: (i) endo-β-1,4-xylanases, which hydrolyze the polysaccharide by attacking internal links in the main chain, releasing oligosaccharides with different degree of polymerization, and (ii) β-xylosidases, which complete the degradation process by converting the xylooligosaccharides into xylose [10].

Although xylanases can be produced by several organisms (including crustaceans, protozoans, snails, insects, bacteria or fungi), filamentous fungi are usually chosen for these studies because they secrete high levels of enzyme (facilitating its purification and further characterization), and for their robustness against environmental factors [11]. Fungi from the genera *Aspergillus, Trichoderma, Rhizopus* and *Penicillium* are very well-known producers of xylanase activities. Specially, *Aspergillus* and *Trichoderma* species have been studied extensively for their ability to secrete high-levels of enzymes in solid-state fermentation (SSF) [12,13,14,15,16]. *Aspergillus* is a genus usually employed to produce xylanolytic enzyme cocktails in industrial plants for applications like paper manufacturing, bread-making, animal feed, juice and wine industries [17,18] and, thus, several studies reporting enzymatic production optimization have been released, either by traditional One-Factor-at-a-Time (OFAT) approach [19] or by Response Surface Methodology (RSM) [20,21]. However, since the enzymatic system secreted by fungi depends on the strain and the culture conditions, it is interesting to study new isolates to produce efficient and robust enzymes.

Solid-state fermentation of lignocellulosic biomass has successfully been applied for biofuels production, for biological detoxification or bioremediation, and for environmental protection by biotransformation of crops and waste products to enhance their nutritional value. These biotransformation processes for crops and waste can be optimized to achieve a suitable high yield of the target product. The classical methods for optimization focus on changing one variable, while maintaining the others fixed. This single-dimensional approach is an incomplete strategy to produce optimized conditions since it does not consider interactions between factors. RSM, on the contrary, is a strong model for establishing optimal process conditions under complex interactions between independent variables [22].

The objective of this study was to optimize the fermentation conditions of a new *A. niger* BG strain growing on wheat straw to produce high levels of β-1,4-endoxylanase, using OFAT and RSM approaches. In addition, this enzyme was purified, its physicochemical and kinetic properties were studied, and a procedure to generate xylooligosaccharides by hydrolysis of wheat straw xylan was designed.

## 2. Results and Discussion

### 2.1. Endoxylanase Optimization Using the Classical OFAT Method

**Figure 1:**
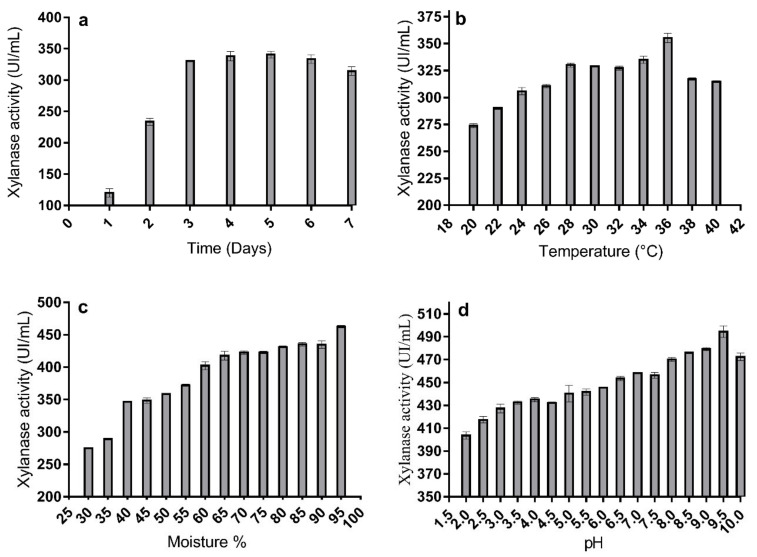
Effect of incubation period (**a**), temperature (**b**), moisture agent level (**c**) and initial pH value (**d**) on the endoxylanase production of *A. niger* BG strain growing under SSF conditions.

There is an increasing interest in endoxylanases since they are essential for biotechnological conversion of xylan into smaller sugars, that have relevant applications as prebiotics, for animal food or to produce fuels [23]. As previously mentioned, *Aspergillus* species are among the most studied organisms in this context, because they are a promising source of robust cellulases and xylanases. *A. niger* BG, identified by its morphological characteristics and the analysis of the ITS region of ribosomal DNA [24], was selected in a previous screening among 62 fungal strains isolated from lignocellulosic wastes by its ability to secrete high levels of enzymes involved in the degradation of plant cell polysaccharide (not shown). The current work analyzes the capabilities of this novel strain to produce an endoxylanase under SSF conditions, with wheat straw as a single carbon source. Under the initial conditions tested, maximal xylanase activity was obtained after seven days of incubation at 28 °C (314.5 ± 0.6 U/mL), when wheat straw was moistened with Mandels solution (70%) at pH 5 and inoculated with a 10^7^ spore suspension/g of substrate. To increase yield, operating conditions such as pH, temperature, moisture level, and incubation time were varied. Figure 1 shows the variations over time of endoxylanase activity in the different tests carried out by using classical OFAT approach, as explained below.

#### 2.1.1. Effect of the Incubation Period

The incubation period is an important parameter when considering enzymatic production. Figure 1a shows the time course of endoxylanase production by *A. niger* BG growing with wheat straw as substrate of SSF. The extracellular activity increased during the first days of incubation, peaking after three days (330.5 ± 0.6 U/mL) and remaining practically stable thereafter. This stabilization could be related to the reduction of nutrients in the fermentation medium along time, which could impact fungal physiology [25]. Similar results have been described in other species of this genus, like *A. fumigatus* F-993 and *Aspergillus flavus*, whose maximum xylanase production was observed after two days of incubation in white corn flour, wheat bran, and pearl millet stover under SSF, respectively [26,27]. On the contrary, maximal production of this activity has been reported for *Rhizopus oryzae* UC2 and *A. fumigatus* MS16 in 4, 5 and 6 day-old cultures with raw oil palm frond leaves, wheat straw, and banana peels as carbon sources, respectively [28,29]. Obtaining high enzyme levels in short incubation times is very interesting for large-scale industrial production, reducing the possibility of contamination and benefiting the economy of the process [26].

#### 2.1.2. Effect of Incubation Temperature

The maximum endoxylanase activity (355.2 ± 0.3 U/mL) was found at 36 °C. According to the literature, the production of xylanase decreases at lower temperature maybe due to lower transfer of substrates in the cell membrane, while higher temperatures could reduce growth due to the denaturation of enzymes, which results in the higher preservation energy for cellular growth and lower metabolites generation [30]. Irfan et al. and Gautam et al. [27,31], registered the maximum xylanase activity at 30 °C by *Aspergillus foetidus*, and *A. flavus* ARC-12. The maximum xylanase production was also reported at 35 °C by *A. fumigatus* MS16 [29] depending on the culture conditions. Temperature heavily influences the SSF process, affecting different parameters as cell growth and production of enzymes and metabolites [32].

#### 2.1.3. Effect of Moisture Level

Moisture affects the physical properties of the solid substrate used for fermentation, influencing enzyme production and microbial growth in the SSF medium [33]. Thus, the optimum moisture level for xylanase production under a given set of SSF conditions should be empirically determined. The data presented in Figure 1c show that moisture increase enhanced endoxylanase secretion by *A. niger* BG, reaching maximum extracellular activity levels at 95% (462.5 ± 0.2 U/mL). Behnam et al. [34] found that 71.8% was the optimum moisture for maximum xylanase production by *R. oryzae*, and Mandal and Ghosh [32], described that the best cellulase production by *A. niger* using banana peel as substrate was obtained with a substrate/liquid ratio of 3:1 (*w*/*v*). More porous substrates can enhance the gaseous transfer, and still retain the right amount of moisture supporting the stability of the extracellular enzyme and nutrient solubility [35]. Conversely, Ezeilo et al., Zehra et al. and Pandey et al. [28,29,36] achieved maximum xylanase production with 40%, 45% and 50% moisture, respectively, in SSF cultures of *R. oryzae* UC2 in raw oil palm frond leaves, *A. fumigatus* MS16 in banana peels, and *R. oryzae* SN5 in wheat bran.

#### 2.1.4. Effect of pH

The pH in a SSF medium is a key variable that directly influences the stability of inter-membrane transportation, microbial growth, and ultimately the production of enzyme. In particular, this is very important for extracellular enzymes, since its release into the medium depends on the mechanism of membrane transport, which is regulated by the hydrogen concentration in the medium [37]. To examine the optimum initial pH for xylanase production, experiments were implemented at different pH values ranging from 2 to 10. Figure 1d shows that xylanase production was quite affected by the initial pH value in the *A. niger* BG cultures growing in wheat straw under SSF conditions. The maximum endoxylanase activity was achieved at pH 9.5 (494.56 ± 0.54 U/mL). Although many fungi exhibit the highest xylanase activity in more or less acidic environments [38], the optimal production of xylanase at pH 8–9 was also reported for *R. oryzae* UC2 [28], *Aspergillus fischeri* [39] and *Trichoderma* SG2 [40]. Some *Aspergillus* have been shown to grow well at pH values between 2–11 [41].

### 2.2. Box-Behnken Analysis of Xylanase Production-Based on RSM Design

The classical approach used to optimize xylanase production by *A. niger* BG on wheat straw indicated that the maximal production was achieved in three days, at alkaline pH (9.5), high humidification level (95%), and mesophilic temperature (36 °C). Under these conditions, *A. niger* BG strain produced high amounts of xylanase with a maximum activity of 494.56 ± 0.54 U/mL (Figure 1d).

The same parameters, temperature (*X*_1_), moisture level (*X*_2_), pH (*X*_3_) and incubation time (*X*_4_), were the independent variables included in a RSM experiment using a Box-Behnken Design (BBD) aimed to predict the conditions that would produce the maximum xylanase yield. Table 1 gathers the experimental design and the levels of variation for each variable around the zero level (the optimum value determined in the OFAT trial), collecting the 27 experimental conditions assayed and the amount of xylanase produced in each particular condition. The maximal xylanase production was achieved in cultures maintained at 36 °C for 96 h with 95% moisture and an initial pH of 11, demonstrating the high tolerance to alkali of this isolate.

#### 2.2.1. Model Performance and Fitting Using RSM

An analysis of variance (ANOVA) allowed us to estimate the significance of the quadratic model built with the experimental data (Table 2). The *F*-value of 12.83 implies that the model is statistically significant (*p*-value < 0.001). Within the model, *X*_1_ (temperature), *X*_2_ (moisture level), *X*_2_*X*_4_ (moisture level vs. incubation time), *X*_2_^2^ (moisture level^2^), and *X*_4_^2^ (incubation time^2^) are significant terms (Table 2) with *p*-values less than 0.05. On the contrary, *p*-values are higher than 5% for factors *X*_3_ (pH), *X*_4_ (incubation time), *X*_1_*X*_2_ (temperature vs. moisture level), *X*_1_*X*_3_ (temperature vs. pH), *X*_1_*X*_4_ (temperature vs. incubation time), *X*_2_*X*_3_ (moisture level vs. pH), *X*_3_*X*_4_ (pH vs. incubation time), *X*_1_^2^ (temperature^2^) and *X*_2_^2^ (moisture level^2^), indicating that these factors and interactions are non-significant. Lack-of-Fit with an *F*-value of 3.36 implies this is not significant relative to the pure error, showing that the model correctly explains the experimental data.

The determination of coefficient value (R^2^) is 0.9374, indicating the acceptability of the model in estimating the predicted values from the experimental data. The model is adequate in explaining most of the variability in the trial results since the R^2^ value is greater than 0.75 [42]. The Adjusted R^2^ value (0.8643) validates the proposed model. The signal-to-noise ratio (Adeq Precision) reflects the precision of the model and its value should be over 4 [43]. The value calculated here was 12.433, which shows a high level of precision and an appropriate response ratio. This value, and that of the coefficient of variation (CV%; 19.9%), suggest that the model is reliable and reproducible [44,45].

**Figure 2:**
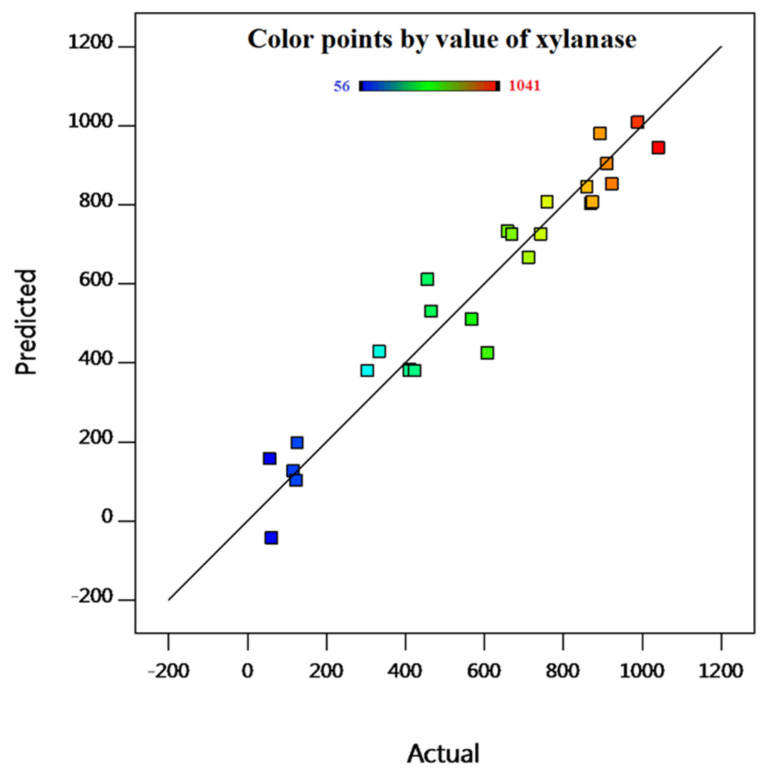
Actual value vs. predicted value split from RSM design.

As observed in Figure 2, the correlation between the values of xylanase production predicted by the RSM and the actual values is good, the linear distribution is indicative of a good-fitted model and, thus, the model is fairly realistic. Since the discrepancy between the actual and predicted values is very small [46], and the R^2^ value of 93.7% confirms the precision of the adjustment, this model can be utilized to sail the design space. Hence, based on the statistical data, it may be concluded that the model is adequate to establish the principal impacts of the factors [47].

The second-order polynomial quadratic regression equation calculated for xylanase production in terms of coded factors (Equation (1)) is:

(1) $$\begin{array}{r} R=+379.67-197.83X_{1}-293.67X_{2}-9.50X_{3}+58.83X_{4}+59.50X_{1}X_{2}+31.75X_{1}X_{3}-18.75X_{1}X_{4}\\ -6.00X_{2}X_{3}+145.00X_{2}X_{4}+79.75X_{3}X_{4}+70.79X_{1}^{2}-79.71X_{2}^{2}+135.54X_{3}^{2}+299.79X_{4}^{2} \end{array}$$

where *R*: Xylanase production (U/mL), *X*_1_: temperature, *X*_2_: moisture level, *X*_3_: pH and, *X*_4_: incubation time. The negative sign before terms indicates an antagonistic effect, and the positive sign indicates a synergistic effect. The development-based model with coded factors is a desirable one since it can help to determine the more significant factors that will have an impact on the response [22].

#### 2.2.2. Analysis of the Interactions between Influencing Factors

**Figure 3:**
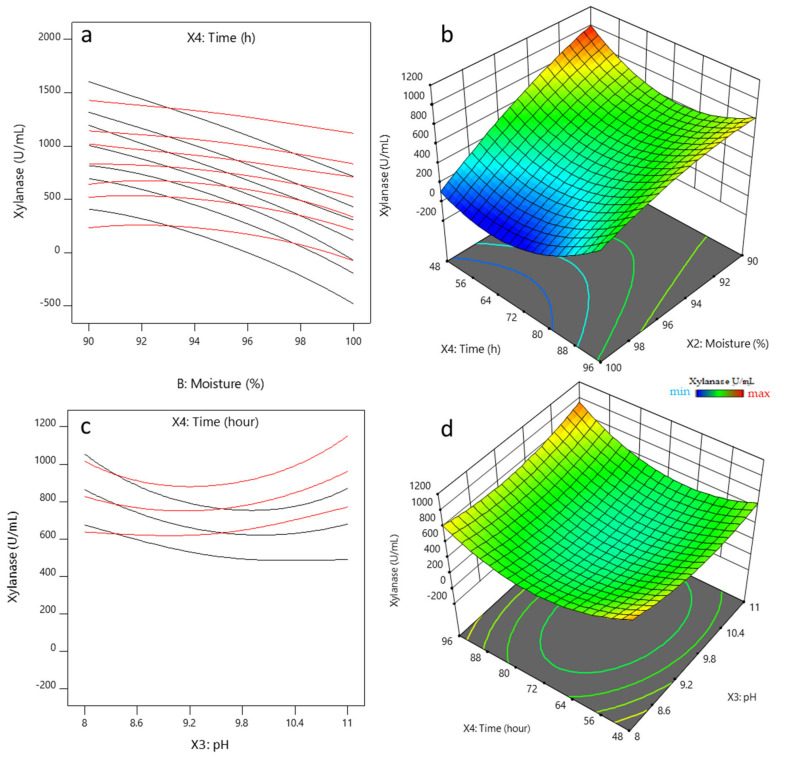
Interaction plots (**a**,**c**) and response surface plot (**b**,**d**) for the model of the intersection between moisture level (*X*_2_) (**a**,**b**) vs. incubation time (*X*_4_) or pH (*X*_3_) (**c**,**d**) vs. incubation time (*X*_4_) for endoxylanase production by *A. niger* BG strain.

The interaction of significant parameters and the effects on response were investigated by interaction plots and three-dimensional response surfaces plots (Figure 3a,b) based on the regression analysis of the BBD. The variation in response (xylanase production) and the significant *p*-value (0.0251) for the interaction between *X*_2_*X*_4_ (moisture level and incubation time) proved the presence of positive interactions between these two variables (Figure 3a). Figure 3b depicts the effect of *X*_2_*X*_4_ on xylanase activity when temperature and initial pH were fixed at level 0. Xylanase activity increased significantly (*p* < 0.05) by increasing the incubation time and the moisture level. However, the production of xylanase mainly depends on the moisture level, as the quadratic and linear effects of this factor were highly significant (*p* < 0.0001), confirming the single-factor experiment results (Table 2) and the influence in the metabolic rate of the interaction between these two parameters. Figure 3c,d shows the effect of *X*_3_*X*_4_ on the xylanase activity. However, this interaction is not significantly influenced by the response at the confidence interval studied (95%). According to studies conducted by Behnam et al. and Cao et al. [45,48], the interaction between incubation time vs. initial pH was insignificant in the optimization of xylanase production by *Mucor indicus* and *Aspergillus niger* AN-13 through SSF and submerged fermentation, respectively. However, Bhardwaj et al. [21] found interaction between pH and xylanase production when *Aspergillus* was grown on rice straw.

Behnam et al. [48] reported that the interaction between moisture and incubation time was significant for xylanase production by *Mucor indicus* and were non-significant for *Mucor hiemalis* and *R. oryzae* xylanase through SSF on wheat bran. In this sense, Tai et al. [20], reported significant interaction between xylanase production and moisture level when *A. niger* DWA8 was grown using agricultural waste. According to studies conducted by Almeida et al. and Azzouz et al. [24,49,50], the interaction between the moisture level and incubation time does not significantly affect biomass and xylanase production by *Penicillium roqueforti* ATCC 10110, *Trichoderma afroharzianum*, and *A. niger* BG during solid-state fermentation of yellow mombin residue and wheat bran, respectively.

To summarize these results, moisture and cultivation time affect xylanase production by *A. niger* BG. The optimum moisture level will depend on the microorganism requirement, type of substrates and end products, and short incubation times provide good conditions for the economical production of enzymes.

#### 2.2.3. Validation of the Model

The optimum values for variables were determined according to the database generated with the software Design Expert 11.6.0. Validation of the statistical model and the regression equation were performed from the optimum conditions proposed by the model, turning a 2 trial run. Table 3 gathers the experimental conditions of the two runs together with the actual xylanase value measured under these conditions. The actual and predicted xylanase production were not significantly different, which allows us to conclude that the model was good at predicting xylanase production.

The optimal values of the four variables were 35 °C (temperature, *X*_1_), 96% (moisture level, *X*_2_), 11 (pH, *X*_3_), and 96 h (incubation time, *X*_4_) and the maximum predicted xylanase activity was 1344 U/mL, a value close to the actual activity of 1413.9 ± 0.2 U/mL. The specific activity of the xylanase produced in the RSM-optimized conditions was calculated as 516.01 U/mg of protein.

The high tolerance to alkali of this isolate of *A. niger* is confirmed again in this experiment. In general, filamentous fungi can grow over a wide pH range with almost constant intracellular pH (in *Aspergillus*, around 7.6), although the changes in the extracellular pH are very stressing conditions for the microorganism. A recent paper from Markina-Iñarrairaegui et al. (2020) describes that *Aspergillus nidulans* becomes adapted to alkaline pH by unchaining a stress response mediated by three main regulatory pathways, and points to the concerted action of two ATPase-like transporters (EnaA and EnaB) as the main responsible for adaptation to alkaline pH [51].

RSM modeling strategies have been used for improving xylanase production by *Aspergillus candidus* [52] and *A. fumigatus* ABK9 [53] with wheat straw as the substrate, as well as other *Aspergillus* strains growing on related substrates [20,21]. Therefore, it may be concluded that BBD-based RSM strategies can significantly enhance xylanase production. The effect of the optimization method is shown by the increased enzyme production and also by the decrease in enzyme production cost, which is mostly based on the use of inexpensive and easily accessible complex substrates like wheat straw and bran [54].

### 2.3. Purification and Physicochemical Properties of A. niger BG Endoxylanase

The xylanase was purified from the crude enzyme extracts showing maximum activity (1413.9 U/mL). After two anion-exchange chromatography steps, a fraction with xylanase activity was purified with a yield of 6.2%. During the process, the specific activity increased from 516.06 to 728.25 U/mg (1.41-fold purification) (Table 4).

**Figure 4:**
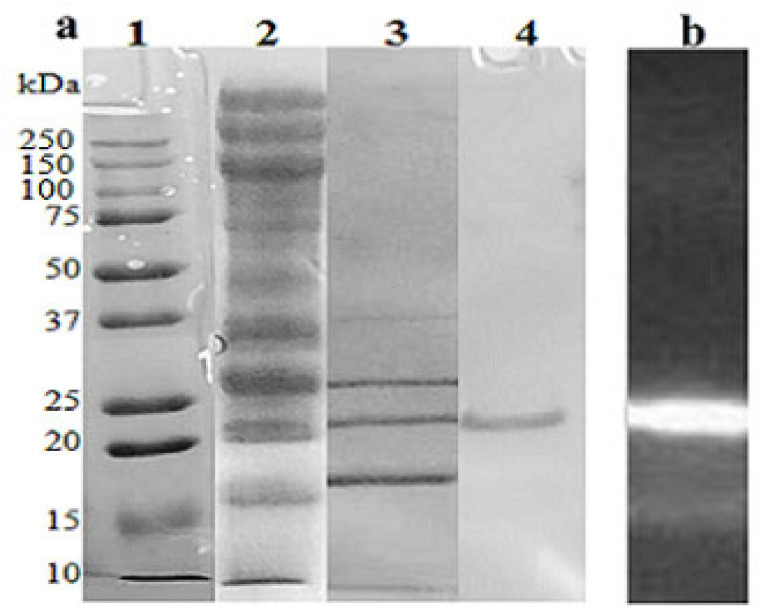
SDS-PAGE (**a**) and zymogram analyses (**b**) of purified *A. niger* BG endoxylanase. (**a**) Lane 1, protein standards; lane 2, crude extract; lane 3, proteins after HiTrap QFF chromatography; lane 4, purified enzyme after Mono-Q column.

The homogeneity of the protein was analyzed by SDS-PAGE, showing a unique protein band with a molecular mass of approximately 20 kDa (Figure 4a). This was also corroborated by zymogram on PAGE, showing a single activity band (Figure 4b). For identification, the purified xylanase was digested with trypsin and analyzed by MALDI-TOF/TOF, and the information was used to run a homology search in the NCBI protein database. The xylanase from *A. niger* BG showed 94% identity to an endoxylanase from *A. niger* (BAO02695.1) with a theoretical molecular mass of 22.671 kDa. This molecular mass is close to those reported in other *A. niger* xylanases, as those from strains DSM 195, BCC14405, IBT-90, CGMCC1067, and IBT-90 [55,56,57].

**Figure 5:**
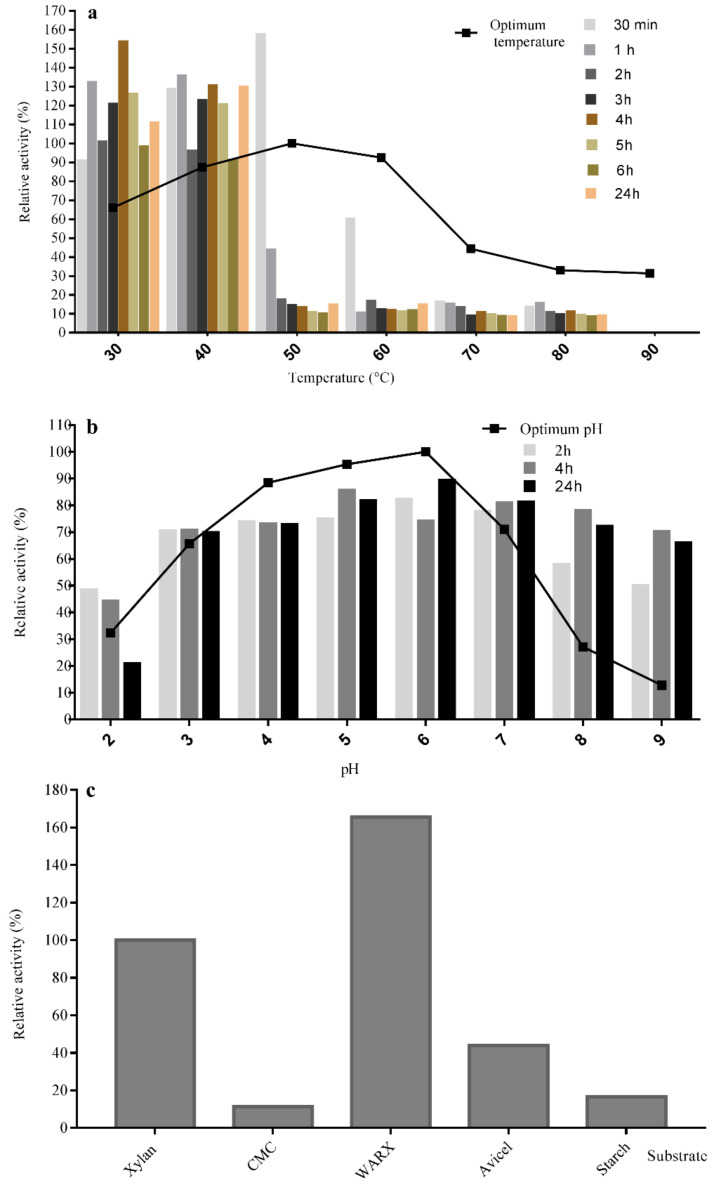
Effect of temperature, pH and different substrates on the purified xylanase activity produced by *A. niger* BG strain. (**a**) Optimum temperature (line) and temperature stability (bars). For optimal temperature, xylanase activity was assayed for 5 min with 1.8% xylan (as described in Section 3.2) but at temperatures between 30–90 °C. For thermal stability analysis, enzyme was incubated at the different temperatures and samples were taken after 0.5, 1, 2, 3, 4, 5, 6, and 24 h for measuring residual activity under standard conditions. (**b**) Optimum pH (line) and pH stability (bars). For optimum pH determination, the initial pH of the reaction was adjusted using different buffers: 50 mM sodium citrate buffer (pH 2.0–5.0), 50 mM sodium phosphate buffer (pH 6.0–8.0) and Tris HCl buffer (pH 9.0–10.0), and activity was measured for 5 min at 50 °C. For pH stability assays, the enzyme was incubated at different pH (using the same buffers) for the indicated time and activity was checked under standard conditions. (**c**) Xylanase specificity towards diverse substrates: the activity was measured at standard assay conditions and the relative activity was calculated for different substrates, considering beech wood xylan activity as 100%. WARX: wheat arabinoxylan.

Regarding the optimum temperature of the pure endoxylanase, (50 °C, Figure 5a) it was similar to that from other enzymes from *A. fumigatus* R1 [58], and *A. nidulans* [59], while those from *A. oryzae* LC1 and *A. niger* XZ-3S had optimal temperatures around 30 and 40 °C, respectively [60,61]. Concerning thermostability, the endoxylanase of *A. niger* BG strain was very stable at 30 and 40 °C, maintaining more than 90% of its activity after 24 h. This is an interesting result, since endoxylanases stable at 40 °C are necessary for xylooligosaccharides production, as well as in baking industry [62]. At higher temperatures, such as 50 and 60 °C, 60% enzyme activity was conserved in the first 30 min, but after 1 h the xylanase activity was partially lost. The stability of the endoxylanase from *A. niger* BG at 30, 40, and 50 °C, as well as its stability in acidic conditions, suggest its suitability for lignocellulosic biomass treatments [63]. Previous studies about the thermostability of xylanases from *Aspergillus* strains indicate that they are generally stable between 35–60 °C [58,61].

The optimum pH and pH stability of the enzyme were also assayed (Figure 5b). The results indicate that the highest xylanase activity is observed at pH 6, being very active between 3–7. The enzyme showed a broad range of pH stability, from 3 to 9 after 24 h incubation at 25 °C, suggesting its ability to act at both acidic and basic pH. Some studies indicate that the optimum pH of xylanases produced by *Aspergillus* species is usually acidic, for example 4.8 for *Aspergillus terreus*, 5.0 for *A. oryzae* LC1, 5.5 for *A. fumigatus* FC2-2, and 6.0 for *A. nidulans* [59,60,64,65]. This feature allows the application of xylanases in the wine industry, mostly in reactors where maceration and fermentation occur concurrently, and pH stability is crucial. In the current study, the purified endoxylanase was stable in a pH range 3–9, similarly to that reported in the main xylanase of *A. oryzae* LC1, expressed in the prokaryotic system *E. coli* [60], and higher than those described for other *A. niger* strains (pH 3.0–7.0) [66].

#### Substrate Specificity and Kinetic Parameters of *A. niger* BG Endoxylanase

The hydrolytic activity of the pure endoxylanase on various substrates is shown in Figure 5c. The results indicate that the enzyme was more active against xylan-containing substrates. The hydrolysis of wheat arabinoxylan (165.7%) widely overpassed that of beechwood xylan (taken as the 100%), a standard substrate for xylanases. Both xylans have high xylose content, but outstanding structural differences. Beechwood xylan has few ramifications, containing approximately 94% linear units of β-1,4-xylose, whereas in wheat arabinoxylan the xylose backbone is highly branched with arabinose residues [67]. In addition, this enzyme acts, although with minor efficiency, on Avicel (43.9%), a polymer of microcrystalline cellulose. The results suggest that the new *A. niger* endoxylanase is a versatile enzyme that acts on linear and branched xylan but also on β-1,4-glucans. However, the endoxylanase from *A. niger* BG is not active on starch, a homogenous α-1,4-glucan, which indicates the enzyme selectively for β-linkages.

The kinetic parameters of the pure xylanase were determined according to the Michaelis-Menten plots, using various concentrations of wheat arabinoxylan as substrate (Table 5). *K*_m_ and *V_max_* values were found to be 26.06 mg/mL and 5,647 U/mg, respectively. The low *K_m_* value indicates a high affinity for the substrate, which is a significant characteristic for industrial processes. The *V_max_* calculated in the present study is relatively high compared to the *V_max_* of xylanases reported for other fungal strains. Thus, the specificity constant (*V_max_*/*K_m_*), that reflects its affinity and catalytic ability [68], is also high.

### 2.4. Xylooligosaccharides Production

**Figure 6:**
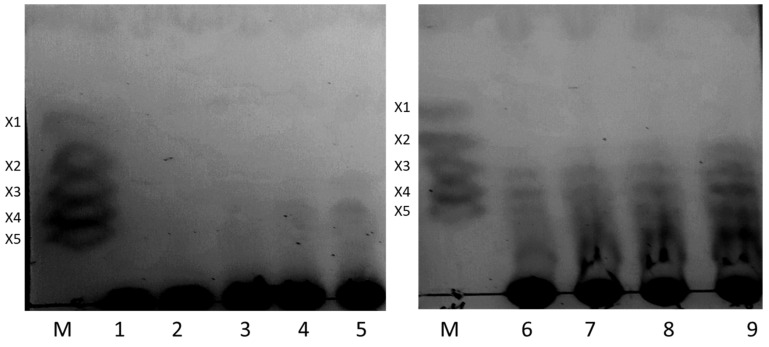
TLC analysis of β-1,4-endoxylanase hydrolysed products from wheat straw xylan. Lanes: M: Markers (X1; xylose, X2; xylobiose, X3; xylotriose, X4; xylotetraose and X5; xylopentaose). 1: Negative control; 2–9: reaction products after 2–5 min, 3–15 min, 4–30 min, 5–1 h, 6–2 h, 7–4 h, 8–6 h, and 9–24 h.

As previously mentioned, xylanases can be used to produce oligosaccharides from xylans. Thus, the purified β-1,4-endoxylanase produced by *A. niger* BG was evaluated for this application using wheat straw xylan as substrate. The hydrolysis mixtures obtained at different reaction times were analyzed by TLC, showing barely visible spots corresponding to xylopentaose, xylotetraose and xylotriose after 15–30 min, that became evident in 1 h reaction samples (Figure 6). Xylobiose spots appeared after 4 h of incubation. According to the results, the concentration of xylooligosaccharides increase as a function of the reaction time. Previous studies have indicated that endoxylanase is the key enzyme for conversion of xylans into xylooligosaccharides [78,79]. The soft and green conditions used for enzymatic production of xylooligosaccharides and the specificity of the biocatalysts make enzymatic methods more convenient than chemical procedures [80], constituting a reasonable and reliable alternative to produce the large amounts required, for example, in aquaculture and poultry industry [81]. Xylooligosaccharides have been qualified as anti-diabetic, anti-hypercholesterolemic, or anti-apoptotic molecules [82], and have been studied as a prebiotic compounds for humans [83], because they are not degraded by the human digestive enzymes, but are fermented into the gastrointestinal tract [84]. According to the results presented here, this β-1,4-endoxylanase produces a high yield of xylooligosaccharides and thus can be a potentially suitable candidate for treatment of biomass.

## 3. Materials and Methods

### 3.1. Microbial Strain and Growth Conditions

*A. niger* BG strain was isolated from olive tree decomposing soil from Akbou area, Bejaia, located in North-East of Algeria [24]. A spore suspension in 1% (*v*/*v*) Tween 80 was prepared from PDA agar plates grown for 7 days. The spore count was performed in a Neubauer counting chamber (Marienfeld, Lauda-Königshofen, Germany) [85]. For solid-state fermentation (SSF) experiments, a 500 mL flask containing 10 g of wheat straw were moistened at 70% with Mandels medium [86], and pH was adjusted to 5.0. This culture medium was sterilized and inoculated (10^7^ spore/g of substrate), and flasks were incubated at 28 °C for 7 days. Thereafter, the enzymatic extracts were harvested by crushing the contents of the flasks in 100 mL of distilled water with a glass rod and then shaking on an orbital shaker at 100 rpm for 10 min at room temperature. The filtrate was centrifuged at 10,000× *g* for 10 min at 4 °C. The clear supernatant was used as crude enzyme with endoxylanase activity and stored at 4 °C until its use. For optimization studies, the composition of the culture medium was varied according to the experimental data, while the inoculum size and source of carbon (wheat straw) were constant.

### 3.2. Enzyme Assay

Endoxylanase activity under standard conditions was measured by the method of Bailey et al. [87] with slight modifications. The liberation of reducing sugars from beech wood xylan was estimated by the dinitrosalicylic acid method [88], using d-xylose as a standard. Reaction mixtures contained 100 µL of properly diluted enzyme with 900 µL of 2% (*w*/*v*) beechwood xylan (Sigma-Aldrich, St. Louis, MO, USA), dissolved in 50 mM citrate buffer (pH 5), and they were incubated for 5 min at 50 °C. Reactions were terminated by the addition of 1.5 mL 3,5-dinitrosalicylic acid reagent (DNS), placed in a boiling water bath for 5 min and cooled in ice water. The absorbance was read at 540 nm. One unit of activity has been defined as the quantity of enzyme needed to release 1.0 μmol of xylose per minute (min) at pH 5 and at 50 °C. For each assay in this study, triplicate measurements were conducted. The concentration of the protein was measured by the method of Bradford [89], using bovine serum albumin (BSA) as a standard.

### 3.3. Determination of Optimal Physicochemical Parameters Using the Classical One-Factor-at-a-Time Approach

The production of endoxylanase from wheat straw under SSF was optimized by classical OFAT approach, that consists on changing one parameter while maintaining the others at predefined levels [47]. In this work, the initial conditions were those stated above: 7 incubation days at 28 °C, in SSF medium at pH 5.0 with 70% moisture. The effects of several physicochemical parameters, culture time (1 to 7 days), temperature (20 to 40 °C), moisture level (30 to 95%) and initial pH value (2 to 10), were analyzed. The initial pH values were obtained by adjusting the initial pH of the medium at desired values with hydrochloric acid (HCl) or sodium hydroxide (NaOH) solutions. All experiments were run in triplicate and the data were subjected to one-way analyses of variance (ANOVA) carried out using XLSTAT software (version 2009.1.02, Addinsoft Inc, New York, NY, USA), determining Fisher’s least significant difference in the mean variable. A *p*-value < 0.05 between the variables was considered significant.

### 3.4. Determination of Optimal Physicochemical Parameters with Response Surface Model Using Box-Behnken Design

Box-Behnken design (BBD) is used for optimization purposes since it allows to estimate the main effects of some factors simultaneously [90]. The parameters and levels used for this optimization (Table 6) were selected by classic OFAT. A polynomial model based on 27 experiments with 3 replicates was built (Table 6) using Design-Expert 11^®^ software (Version11.0.5.0., Statease, Minneapolis, MN, USA).

The number of experiments (*N*) required was defined according to Equation (2):

(2) $$N=2k\cdot (k-1)+C0_{,}$$

where *k* and *C*_0_ are the number of factors and central points, respectively.

The trial data are then fitted through the response surface model (RSM) to discover the relationship between the controllable factors and the response factors [91]. Regression analysis of the data to fit a second-order polynomial equation (quadratic model) was carried out according to the following general Equation (3), which was used to predict the optimum conditions for enzyme production.

(3) $$R=\beta 0+\sum_{i=1}^{n}\beta iXi^{}+\sum_{i=1}^{n}\beta iiXi^{2}+{\sum_{i=1}^{n}\sum_{j=1}^{n}\beta ijXiXj+\varepsilon }_{,}$$

where *R* represents the response surfaces, *β*_0_ is the constant term (intercept), *β_i_, β_ii_* and *β_ij_* are the linear, quadratic, and interaction coefficients, respectively, while *X_i_*, *X*^2^ and *X_i_X_j_* are linear variables, quadratic variables, and term for interaction of the variables respectively, and *ε* is the residual associated to the experiments (the prediction error, represents the difference between measured and predicted R values and quantifies the random variability in our experimental design). The surface plots were obtained by varying the values of two factors, keeping the values of other factors constant at the zero level. The second-order polynomial model developed by RSM consists of linear, quadratic, and interaction models (Equation (3)) [92]. The determination coefficient (*R*^2^) measures the goodness of fit (Equation (4)). The closer the value is to 1, the better the model fits.

(4) $$R^{2}=1-{\left[\sum_{i=1}^{n}{\left(R_{i}-\overset{⏜}{R_{i}}\right)}^{2}÷\sum_{i=1}^{n}{\left(R_{i}-{\bar{R}}_{}\right)}^{2}\right]}_{,}$$

where *n* is the number of measures, *R_i_* the *i*-th observation value, $\overset{⏜}{R_{i}}$ the *i*-th predicted value, *i* the number of trials and ${\bar{R}}_{}$ the average of the response factors.

The predicted determination coefficient (*R*^2^*_predict_*) indicates the predictability of the regression model as given in Equation (5).

(5) $$R^{2}{}_{predict}=1-\left(1-R^{2}\right)\left[\left(n-1\right)÷\left(n-\left(k+1\right)\right)\right]$$

where *n* is the number of observations and *k* is the number of independent variables in the regression equation. The closer *R*^2^*_predict_* is to 1, the better the predictability of the model is. The model is simplified by rounding off the non-significant items in the model, obtaining a new RSM model with better predictability [91].

### 3.5. Purification of β-1,4-Endoxylanase

The optimal SSF conditions obtained after RSM optimization were used to grow *A. niger* BG for enzyme production. The crude extract (500 mL) was recovered and centrifuged for 15 min at 10,000× *g* to remove mycelium and spore cells. The supernatant was sequentially filtered, using 0.8, 0.45 and 0.22 μm filter membranes (Merck-Millipore, Kenilworth, NJ, USA) and then concentrated and dialyzed against 10 mM sodium phosphate buffer pH 6.0 by ultrafiltration, using a 3-kDa cut-off membrane. All purification steps were performed using an ÄKTA purifier system (GE Healthcare, Chicago, IL, USA). Initially, this crude extract was applied to a 5 mL-HiTrap QFF (GE Healthcare) cartridge, pre-equilibrated with the protein dialysis buffer, at a flow rate of 3 mL/min. Elution was carried out by applying a linear gradient of 1 M NaCl, prepared in the same buffer, from 0 to 50% in 45 min. The protein profile was followed by the absorbance at 280 nm. Fractions with endoxylanase activity were pooled, dialyzed in the buffer mentioned above and concentrated by ultrafiltration using 3-kDa cut-off Amicon Ultra-15 centrifugal devices (Merck-Millipore). Finally, the samples were applied into a Mono Q 5/50 GL (GE Healthcare), equilibrated in the same buffer at a flow rate of 1 mL/min, eluting the proteins by applying a linear gradient of 1 M NaCl (0 to 10% for 35 min). The fractions with the purified protein were pooled, concentrated by ultrafiltration as previously explained, and stored at 4 °C.

### 3.6. Characterization of β-1,4-Endoxylanase

#### 3.6.1. Polyacrylamide Gel Electrophoresis, Mass Spectrometry and NH_2_ -Terminal Amino-Acid Sequencing

The homogeneity and molecular mass of the purified enzyme were determined by sodium dodecyl sulfate-polyacrylamide gel electrophoresis (SDS-PAGE) following the method of Laemmli [93], using 12% polyacrylamide gel and Precision Plus protein dual-color standards (Bio-Rad, Hercules, CA, USA). Proteins were stained with Coomassie brilliant blue R-250 (Sigma-Aldrich).

The native-PAGE (non-reducing conditions) of the purified xylanase was performed using a 12% resolving gel. Xylanase activity was detected according to the protocol described by Yan et al. [94] with slight modifications. Once the electrophoresis was finished, the gel was washed twice (each for 10 min) with 20 mL of 10 mM phosphate buffer, pH 6.0. The treated gel was then pre-incubated in 20 mL of the buffer containing 10 mM xylanase substrate (EnzChek™ Ultra Xylanase Assay Kit, Invitrogen™, Waltham, MA, USA), followed by incubation for 10 min at room temperature. Finally, the detection of xylanase activity was visualized by fluorescence under ultraviolet light using the Gel Doc XR+ system (Bio-Rad). The peptides generated by tryptic digestion of the protein were analyzed by matrix-assisted laser desorption ionization-time of the flight mass spectrometry (MALDI-TOF/TOF) in an Autoflex III instrument (Bruker Daltonics, Billerica, MA, USA). For identification, a combined data search (peptide mass fingerprint and sequence of some of the peptides) in the NCBI database was performed, using the MASCOT engine (Matrix Science, Chicago, IL, USA).

#### 3.6.2. Effect of pH and Temperature on Enzyme Activity and Stability

The optimum pH of the endoxylanase was investigated at pH values ranging from 2 to 10 at 50 °C for 5 min, using 1.8% beechwood xylan as substrate and the following buffers: 50 mM sodium citrate buffer (pH 2.0–5.0), 50 mM sodium phosphate buffer (pH 6.0–8.0) and 50 mM Tris HCl buffer (pH 9.0–10.0). The optimum temperature was measured using standard conditions but at temperatures ranging (30–90 °C). In these experiments, the relative activity was calculated considering the maximum activity as the 100%.

For pH stability tests, enzyme was incubated at 25 °C in the same buffers described above (2.0 to 10.0) and samples were taken after 30 min, 2, 4 and 24 h for standard activity assay. The thermostability of the xylanase was determined by incubating the purified enzyme at 30–90 °C in 50 mM sodium citrate buffer, pH 5.0 in the absence of substrate, and the residual enzymatic activity was determined under standard conditions (pH 5.0, time 5 min and temperature 50 °C) in samples taken after 0.5, 1, 2, 3, 4, 5, 6 and 24 h. In stability tests, initial activity was taken as the 100%.

#### 3.6.3. Substrate Specificity and Determination of Kinetic Parameters

Substrate specificity was investigated by incubating the purified enzyme with beechwood xylan, wheat arabinoxylan (WARX), Avicel (microcrystalline cellulose), carboxymethyl cellulose (CMC) and starch, detecting the release of reducing sugars by the DNS method. The activity was calculated relative to that experimentally calculated for beechwood xylan, that was taken as the reference.

The kinetic parameters (*K_m_* and *V_max_*) of xylanase towards WARX were determined using substrate concentrations from 2 to 50 mg/mL. The data were fitted to a Michaelis−Menten equation using PRISM 7 software (version 7.00, GraphPad, San Diego, CA, USA).

## 4. Wheat Straw Xylan Hydrolysis Using β-1,4-Endoxylanase

The hydrolysis products released by enzymatic hydrolysis of wheat straw xylan, kindly provided by Andrómaco Laboratories (Madrid, Spain), were analyzed by thin-layer chromatography (TLC). 500 μL of substrate (20 mg/mL) were mixed with 0.03 U of pure enzyme in a 1.5-mL tube. The mixtures were incubated at 40 °C for 5 min, 15 min, 30 min, 1 h, 2 h, 4 h, 6 h and 24 h. The samples were centrifuged at 14,000× *g* for 5 min, and the supernatant was used to analyze the hydrolysis products by TLC. For every sample, 5 μL of the hydrolysis mixture were spotted onto TLC plates (POLYGRAM^®^ SIL G/UV_254_, Macherey-Nagel, Germany) and developed with butanol/acetic acid/water (3:1:1, *v*/*v*/*v*). The TLC plates were air-dried for 15–30 min and visualized by spraying with methanol/sulfuric acid (95:5, *v*/*v*), followed by heating at 110 °C for 10 min. A xylooligosaccharides’ solution (xylose, xylobiose, xylotriose, xylotetraose, and xylopentaose) was used as standard.

## 5. Conclusions

The production of xylanase by *A. niger* BG by solid state fermentation of wheat straw was statistically optimized using a classical OFAT approach and RSM, confirming that enzyme yields are highly dependent on moisture. The purified enzyme was very stable in a large range of pH (3–9) and temperature (30–60 °C), and had high hydrolytic activity towards wheat arabinoxylan, successfully producing oligosaccharides from this substrate. These results suggest that it could be of interest for biotechnological processes.

## Figures and Tables

**Table 1:** BBD for 27 runs of experiments.

Run	Factor 1 *X*_1_: Temperature (°C)	Factor 2 *X*_2_: Moisture (%)	Factor 3 *X*_3_: pH	Factor 4 *X*_4_: Time (h)	Response Xylanase Activity (U/mL)
1	40	95	11	72	334 ± 0.18
2	32	95	8	72	874 ± 0.03
3	40	90	9.5	72	608 ± 0.03
4	36	95	8	48	860 ± 0.01
5	40	95	9.5	48	465 ± 0.01
6	36	95	9.5	72	304 ± 0.1
7	36	95	11	96	1041 ± 0.05
8	40	95	8	72	411 ± 0.21
9	36	90	9.5	96	759 ± 0.03
10	32	95	9.5	48	923 ± 0.06
11	36	90	9.5	48	894 ± 0.46
12	36	95	9.5	72	424 ± 0.05
13	36	95	9.5	72	411 ± 0.05
14	32	100	9.5	72	126 ± 0.25
15	36	100	9.5	48	123 ± 0.05
16	36	95	8	96	870 ± 0.01
17	32	95	11	72	670 ± 0.28
18	36	95	11	48	712 ± 0.33
19	40	95	9.5	96	456 ± 0.05
20	36	90	11	72	743 ± 0.38
21	36	100	11	72	116 ± 0.01
22	40	100	9.5	72	61 ± 0.30
23	36	100	9.5	96	568 ± 0.01
24	32	90	9.5	72	911 ± 0.36
25	36	100	8	72	56 ± 0.02
26	36	90	8	72	659 ± 0.30
27	32	95	9.5	96	989 ± 0.42

**Table 2:** ANOVA for quadratic model.

Source	Sum of Squares	DF ^a^	Mean of Squares	*F*-Value	Prob > F	
Model	2.309 × 10^6^	14	1.650 × 10^5^	12.83	<0.0001	Significant
Linear						
*X*_1_-Temperature	3.881 × 10^5^	1	3.881 × 10^5^	30.18	0.0001	
*X*_2_-Moisture	1.035 × 10^6^	1	1.035 × 10^6^	80.47	<0.0001	
*X*_3_-pH	1083.00	1	1083.00	0.0842	0.7766	
*X*_4_-Time	41,536.33	1	41,536.33	3.23	0.0975	
Interaction						
*X*_1_ *X*_2_	14,161.00	1	14,161.00	1.10	0.3147	
*X*_1_ *X*_3_	4032.25	1	4032.25	0.3135	0.5858	
*X*_1_ *X*_4_	1406.25	1	1406.25	0.1093	0.7466	
*X*_2_ *X*_3_	144.00	1	144.00	0.0112	0.9175	
*X*_2_ *X*_4_	84,100.00	1	84,100.00	6.54	0.0251	
*X*_3_ *X*_4_	25,440.25	1	25,440.25	1.98	0.1849	
Quadratic						
$X_{1}^{2}$	26,727.79	1	26,727.79	2.08	0.1750	
$X_{2}^{2}$	33,884.90	1	33,884.90	2.63	0.1305	
$X_{3}^{2}$	97,981.56	1	97,981.56	7.62	0.0173	
$X_{4}^{2}$	4.793 × 10^5^	1	4.793 × 10^5^	37.27	<0.0001	
Residual	1.543 × 10^5^	12	12,860.26			
Lack of Fit	1.457 × 10^5^	10	14,565.05	3.36	0.2511	Not significant
Pure Error	8672.67	2	4336.33			
Cor Total	2.4604 × 10^6^	26				
Model Summary Statistics						
Std. Dev.	113.40	R^2^		0.9374		
Mean	569.19	Adjusted R^2^		0.8643		
CV%	19.92	Adeq Precision		12.4331		

^a^ Degree of Freedom.

**Table 3:** Validation of quadratic model for xylanase production with *A. niger* BG strain optimized by RSM.

Solutions Number	Temperature (°C)	Moisture Level (%)	pH	Incubation Time (h)	Xylanase Predicted (U/mL)	Xylanase Actual (U/mL)	Desirability
1	35	96	11	96	1344	1413.9 ± 0.2	1
2	33	90	9	48	911	824.5 ± 0.6	1

**Table 4:** Purification steps of xylanase from *A. niger* BG strain.

Purification Step	Protein (mg/mL)	Activity (U/mL)	Specific activity (U/mg)	Purification (Fold)	Yield (%)
Supernatant	2.74	1413.88	516.01	1.00	100.00
Ultrafiltration	1.95	1065.74	546.53	1.06	75.38
Hitrap QFF	0.52	506.93	974.88	1.89	36.83
Mono Q	0.12	87.39	728.25	1.41	6.20

**Table 5:** Comparative data of native xylanase from different fungal strains.

Enzyme	Source	Activities U/mg	Optimum pH	Optimum T (°C)	pH Stability	Thermal Stability (°C)	Molecular Weight (kDa)	*K_m_* (mg/mL)	*V_max_* (U/mg)	*V_max_*/*K_m_*	References
Native	*A. niger* BG strain ^a^	728.25	6.0	50	3.0–9.0	30–60	20	26.06	5,647	216.69	This study
xyn11B	*A. niger* BCC14405 ^b^	317	5	55	5.0–10	50	21	8.9	11.1	1.24	[69]
xyn11A	*A. pullulans* Y-2311-1 ^b^	2.240	4.8	54	3.8–5.4	50	25	7.6	2,650	348.68	[70]
xyn11A	*F. oxysporum* f. sp. *lycopersici* F3 ^c^	331	6	60	4.0–10	//	21	0.41	//		[71]
xyn11A	*Paecilomyces thermophila* ^b^	1180	7	75–80	6.0–11	75	25.8	1.6	1424	890	[72]
xyn11C	*P. occitanis* P0I6 ^c^	358	3	45	2.0–10	45	22	14.13	806.3	57.06	[73]
xyn11A	*Penicillium* sp. 40 ^c^	1250	2	50	2.0–5.0	30	25	8.3	6,100	734.94	[74]
xyn11A	*S. commune* *Delar*/ATCC 38548 ^d^	15	5	45–50	6.0–8.0	//	33	8.37	0.443	0.052	[75]
xyn11B	*T. funiculosus* IMI134756 ^b^	196	3.7–4.7	//	//	//	27.84	22	506	23	[76]
xyn11A	*Trichoderma sp.* ^b^	426	6	42.5	3.5–9.0	35	20.5	2.1	//		[77]

Substrate added to SSF: ^a^ wheat straw, ^b^ birchwood xylan, ^c^ oat spelt xylan, ^d^ soluble larch xylan.

**Table 6:** Independent variables and levels of variation in Box-Behnken design (BBD).

Study Type	Response Surface					
Design Type	Box–Behnken		Runs No Blocks		27	
Design Mode	Quadratic					
Factor	Name	Units	Type	Level (−1)	Level (+1)	Level (0)
*X*_1_	Temperature	°C	Numeric	32	40	36
*X*_2_	Moisture	%	Numeric	90	100	95
*X*_3_	pH		Numeric	8	11	9.5
*X*_4_	Time	hour	Numeric	48	96	72
Response	Name	Units	Analysis	Polynomial		
R	Xylanase	U/mL				

## Data Availability

Data is contained within the article.

## Sample Availability

Samples of the compounds are not available from the authors.
